# Inhibition of NMDA receptors and other ion channel types by membrane-associated drugs

**DOI:** 10.3389/fphar.2025.1561956

**Published:** 2025-04-30

**Authors:** Elizabeth G. Neureiter, M. Quincy Erickson-Oberg, Aparna Nigam, Jon W. Johnson

**Affiliations:** Department of Neuroscience and Center for Neuroscience, University of Pittsburgh, Pittsburgh, PA, United States

**Keywords:** NMDAR, MCI, ketamine, membrane, memantine, hydrophobic, channel block

## Abstract

N-methyl-D-aspartate receptors (NMDARs) are ligand-gated ion channels present at most excitatory synapses in the brain that play essential roles in cognitive functions including learning and memory consolidation. However, NMDAR dysregulation is implicated in many nervous system disorders. Diseases that involve pathological hyperactivity of NMDARs can be treated clinically through inhibition by channel blocking drugs. NMDAR channel block can occur via two known mechanisms. First, in traditional block, charged drug molecules can enter the channel directly from the extracellular solution after NMDAR activation and channel opening. Second, uncharged molecules of channel blocking drug can enter the hydrophobic plasma membrane, and upon NMDAR activation the membrane-associated drug can transit into the channel through a fenestration within the NMDAR. This membrane-associated mechanism of action is called membrane to channel inhibition (MCI) and is not well understood despite the clinical importance of NMDAR channel blocking drugs. Intriguingly, a hydrophobic route of access for drugs is not unique to NMDARs. Our review will address inhibition of NMDARs and other ion channels by membrane-associated drugs and consider how the path of access may affect a drug’s therapeutic potential.

## 1 Introduction

**FIGURE 1:**
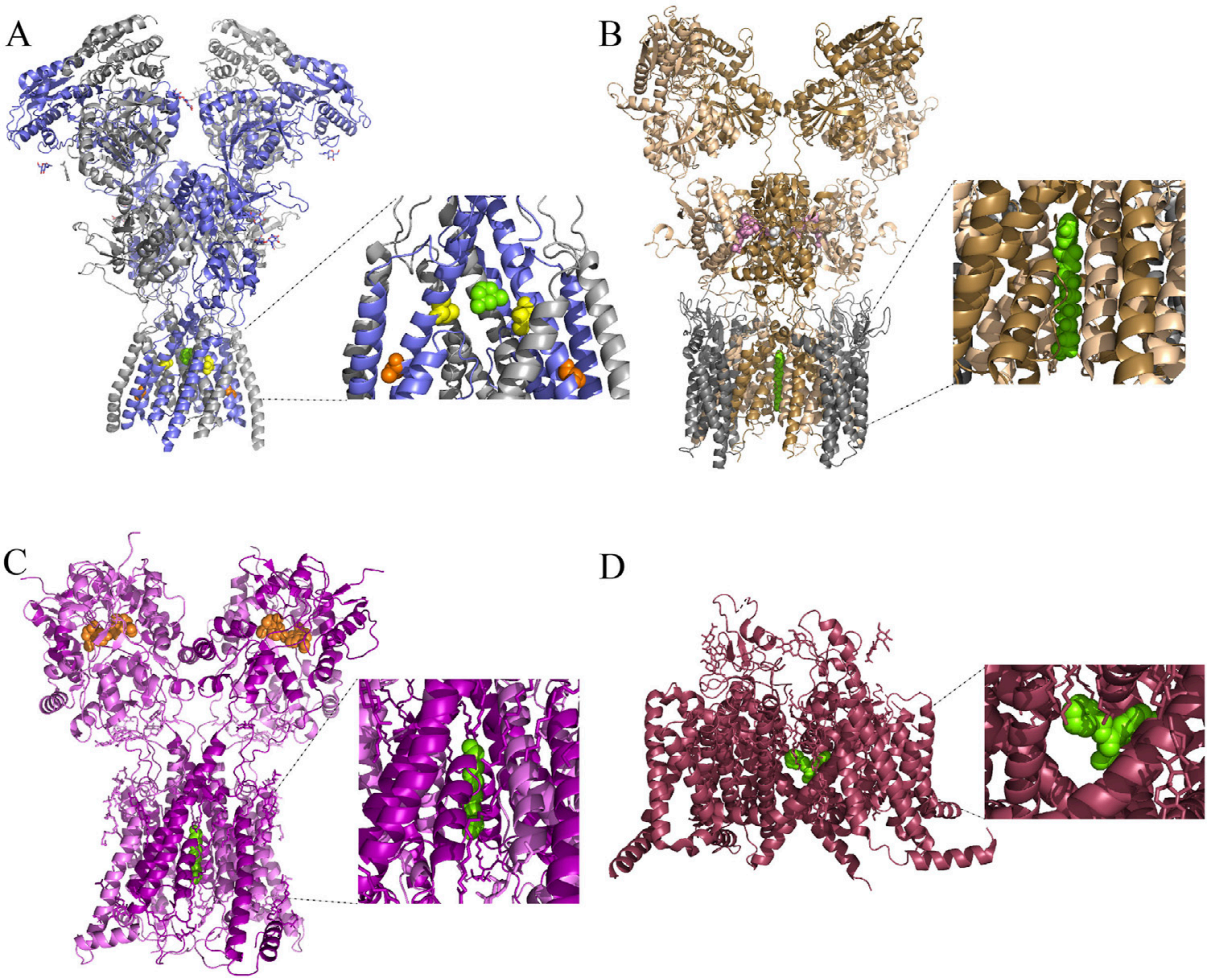
Cryo-EM structures of ionotropic glutamate receptors and a VGSC with channel blocking drugs bound. **(A)** Side view of the structure of an open *rattus norvegicus* (rat) GluN1/2B receptor in complex with the channel blocking drug memantine (green). Inset shows magnified view of bound memantine. GluN1 subunits are in gray, and GluN2B subunits are in blue. A portion of the pre-M4 and M4 regions on the front GluN2B subunit, and of the M3 region on the front GluN1 subunit, have been removed in the inset for visual clarity of the channel blocking site. GluN2A (M630) is a residue that lines the fenestration used by memantine during MCI to access the channel blocking site (Wilcox et al., 2022); the homologous residue GluN2B(M631) is highlighted in orange. There is evidence for at least one additional NMDAR fenestration that connects the central vestibule to the plasma membrane bilayer (Song et al., 2018), although whether this fenestration is involved in MCI is unknown. One of the residues that lines this pathway in *Xenopus laevis* NMDARs is GluN2B(V637). The analogous residue in rat NMDARs is GluN2B(V640), which is highlighted here in yellow. Protein Data Bank (PDB): 7SAD (Chou et al., 2022). **(B)** Side view of the structure of an open homomeric rat GluA2 receptor in complex with stargazin (gray), glutamate (white), cyclothiazide (pink), and the channel blocker NASPM (green). The receptor was composed of four GluA2 subunit-stargazin fusion proteins. The A and C subunits are colored in wheat; the B and D subunits are colored in sand. PDB: 6DM1 (Twomey et al., 2018). **(C)** Side view of the structure of a homomeric rat GluK2 receptor in complex with positive allosteric modulator BPAM-344 (orange) and the channel blocker spermine (green). The A and C subunits are colored in magenta; the B and D subunits are colored in pink. PDB: 9DXS (Gangwar et al., 2024). **(D)** Side view of a rat Na_v_1.5 receptor in complex with the channel blocker flecainide (green). PDB: 6UZ0 (Jiang et al., 2020).

Nervous system function is made possible through neuronal communication. Communication between neurons depends on both voltage-gated ion channels, which typically are activated by membrane depolarization, and ligand-gated ion channels, which typically are activated by binding of neurotransmitter molecules. Activation of voltage-gated and of ligand-gated ion channels leads to opening of a pore through which ions can enter or exit the neuron, affecting membrane potential and changing intracellular ion concentrations. Given the ubiquity of ion channels, their dysfunction can lead to an array of debilitating medical conditions. Channel function can be altered by a wide variety of naturally occurring and synthetic molecules, some of which are useful across a broad range of medical applications. There are many ways that drugs can alter channel activity. This review will focus on drugs that act as channel blockers, which physically inhibit ion flow by binding within the ion conducting pore of a channel. Channel blocking drugs have been described for many types of ion channels; examples of recently published structures of several of the ion channels addressed in this review with a channel blocker bound in its pore are shown in Figure 1. We will explore here hydrophobic paths of access used by inhibitory drugs that act on N-methyl-D-aspartate receptors (NMDARs), α-amino-3-hydroxy-5-methyl-4-isoxazolepropionate receptors (AMPARs), kainate receptors (KARs), nicotinic acetylcholine receptors (nAChRs), γ-aminobutyrate type A receptors (GABA_A_Rs), transient receptor potential (TRP) channels, voltage-gated sodium channels (VGSCs), voltage-gated potassium (K_V_) channels, and voltage-gated calcium channels (VGCC).

## 2 Introduction to NMDARs

N-methyl-D-aspartate receptors (NMDARs) are ionotropic glutamate receptors present at most excitatory synapses in the nervous system. They conduct sodium (Na^+^), potassium (K^+^), and calcium (Ca^2+^) ions across neuronal membranes to transmit neural signals and are extremely important in learning and memory (Abbott and Nelson, 2000; Voglis and Tavernarakis, 2006; Citri and Malenka, 2008). In addition to glutamate, NMDARs require binding of a second type of agonist (commonly glycine or D-serine) to activate. Substantial NMDAR-mediated ion flux requires postsynaptic depolarization to relieve voltage-dependent block of the channel by magnesium (Mg^2+^) in addition to agonist binding (Ault et al., 1980; Mayer et al., 1984; Nowak et al., 1984).

There are seven NMDAR subunits (GluN1, GluN2A, 2B, 2C, 2D, 3A, and 3B), each encoded by a distinct gene. Each subunit contains an extracellular amino-terminal domain (ATD) and ligand binding domain (LBD), a transmembrane domain that consists of the M1, M3, and M4 transmembrane helices and the M2 re-entrant loop, and an intracellular C-terminal domain (Traynelis et al., 2010; Karakas and Furukawa, 2014).

There are many distinct NMDAR subtypes. Functional receptors must be a heteromeric combination of four subunits. Two subunits must be GluN1, and the other two can be any combination of GluN2A-2D and/or GluN3A-3B. NMDARs can be either diheteromeric, formed by two different types of subunits, or triheteromeric, formed by three different types of subunits. Triheteromeric NMDARs are likely the most common type of endogenous NMDAR (Luo et al., 1997; Al-Hallaq et al., 2007; Gray et al., 2011; Rauner and Kohr, 2011; Tovar et al., 2013; Zhang et al., 2025). In adult rat cerebral cortex and hippocampus, GluN1/2A/2B receptors, GluN1/2B receptors, and GluN1/2A receptors were resolved by cryo-EM in a 9:7:4 ratio (Zhang et al., 2025). Each subunit combination has subtype-specific characteristics pertaining to maximal open probability, agonist affinity, gating kinetics, single channel conductance, and selective permeability (Paoletti et al., 2013; Wyllie et al., 2013; Glasgow et al., 2015). For example, the GluN2A subunit has the lowest affinity for glutamate, while GluN2D has the highest (Hollmann and Heinemann, 1994; Hansen et al., 2014). The expression of different NMDAR subtypes varies across brain regions as well as across developmental time. During the second week after birth, the most common GluN2 NMDAR subunit changes from GluN2B to GluN2A (Wyllie et al., 2013). Developmental changes in the surface expression of NMDAR subtypes may depend in part on differential regulation of NMDAR trafficking by the co-agonists glycine and D-serine (Ferreira et al., 2017).

While NMDAR activity is a vital component of healthy neural signaling, NMDAR hyperactivity can result in cell death due to pathologically high levels of intracellular Ca^2+^. Unregulated NMDAR-mediated Ca^2+^ influx and resulting excitotoxicity has been implicated in a wide variety of disease states, including Alzheimer’s disease (AD), chronic pain, Parkinson’s disease, and cell death after stroke (Petrenko et al., 2003; Olivares et al., 2012; Heresco-Levy et al., 2013; Wang and Reddy, 2017; Cappelli et al., 2022). Thus, treatment of NMDAR hyperactivity is of major clinical interest.

### 2.1 NMDAR channel blocking drugs

Pathological NMDAR hyperactivity can be treated using NMDAR channel blocking drugs. This class of drugs inhibits NMDAR activity by binding within the ion channel (Figure 1A), near the location referred to as the N-site to physically inhibit ion flux (Kashiwagi et al., 2002; Chen and Lipton, 2005; Zhang Y. et al., 2021; Chou et al., 2022). The N-site, so named for uncharged asparagine (N) residues located at the extracellular entrance to the narrow selectivity filter, contributes to NMDAR permeation properties (Burnashev et al., 1992; Sather et al., 1994).

Despite sharing a primary mechanism of action, NMDAR channel blocking drugs have a range of distinct clinical effects (Rogawski, 2000; Phillips et al., 2020). For example, memantine is the only NMDAR channel blocking drug approved by the FDA for the treatment of AD and is generally well tolerated. Phencyclidine (PCP) and ketamine are both NMDAR channel blocking drugs that act as dissociative anesthetics and are widely abused due in part to their hallucinogenic effects (Liu et al., 2016; Wallach and Brandt, 2018). The S (+) enantiomer of ketamine (esketamine, sold as Spravato) has been approved by the FDA for the treatment of major depressive disorder (MDD) (Swainson et al., 2019). Ketamine is unique amongst antidepressants because of its rapid action; whereas selective serotonin reuptake inhibitors (SSRIs) take weeks to months to become fully effective, ketamine’s antidepressant effects occur within hours (Yang et al., 2018; Ruberto et al., 2020). Besides binding to NMDARs, ketamine also has affinity at clinically relevant concentrations for hyperpolarization-activated cyclic nucleotide-gated (HCN) channels, as well as opioid, aminergic, and cholinergic receptors (Chen et al., 2009; Mion and Villevieille, 2013; Zorumski et al., 2016; Zanos et al., 2018) which may contribute to its clinical effects. In addition to blocking the NMDAR channel, ketamine may act allosterically at a site accessible from within the plasma membrane (Orser et al., 1997; Abbott et al., 2024). Furthermore, ketamine has several metabolites with additional effects (Zanos et al., 2016). Dextromethorphan and its metabolite dextrorphan are antitussive agents, although both produce hallucinogenic effects at high doses (McClure and Daniels, 2023). Amantadine is another clinically relevant NMDAR channel blocking drug that is used to treat Parkinson’s disease (Kornhuber et al., 1991; Lupp et al., 1992; Blanchet et al., 2003). Although these drugs are all NMDAR channel blockers, the specific molecular mechanisms underlying the range of distinct clinical effects they induce remain poorly understood.

Not all NMDAR channel blocking drugs are of clinical utility. MK-801 is a channel blocking drug with extremely high NMDAR affinity; however, it was found (along with several other NMDAR channel blocking drugs) to be neurotoxic in rat cerebral cortex (Olney et al., 1989) and is not in use clinically. Despite its clinical failure, MK-801 is extremely useful experimentally because of its high affinity for NMDARs. One common experimental application is to add MK-801 to the intracellular recording solution used for whole-cell electrophysiological experiments to selectively inhibit NMDARs on the cell from which recordings are made (Berretta and Jones, 1996; Humeau et al., 2003; Lien et al., 2006; Corlew et al., 2007; Sun et al., 2018).

Although it is unclear why the clinical effects of NMDAR channel blocking drugs are so diverse, the explanation is likely to involve differences in the NMDAR subpopulations the blockers preferentially inhibit (Parsons et al., 1999; Blanpied et al., 2005; Phillips et al., 2020). Further investigation of the mechanisms of action of these drugs may help inform development of improved treatments for NMDAR-associated illnesses.

### 2.2 Routes of NMDAR channel block

**FIGURE 2:**
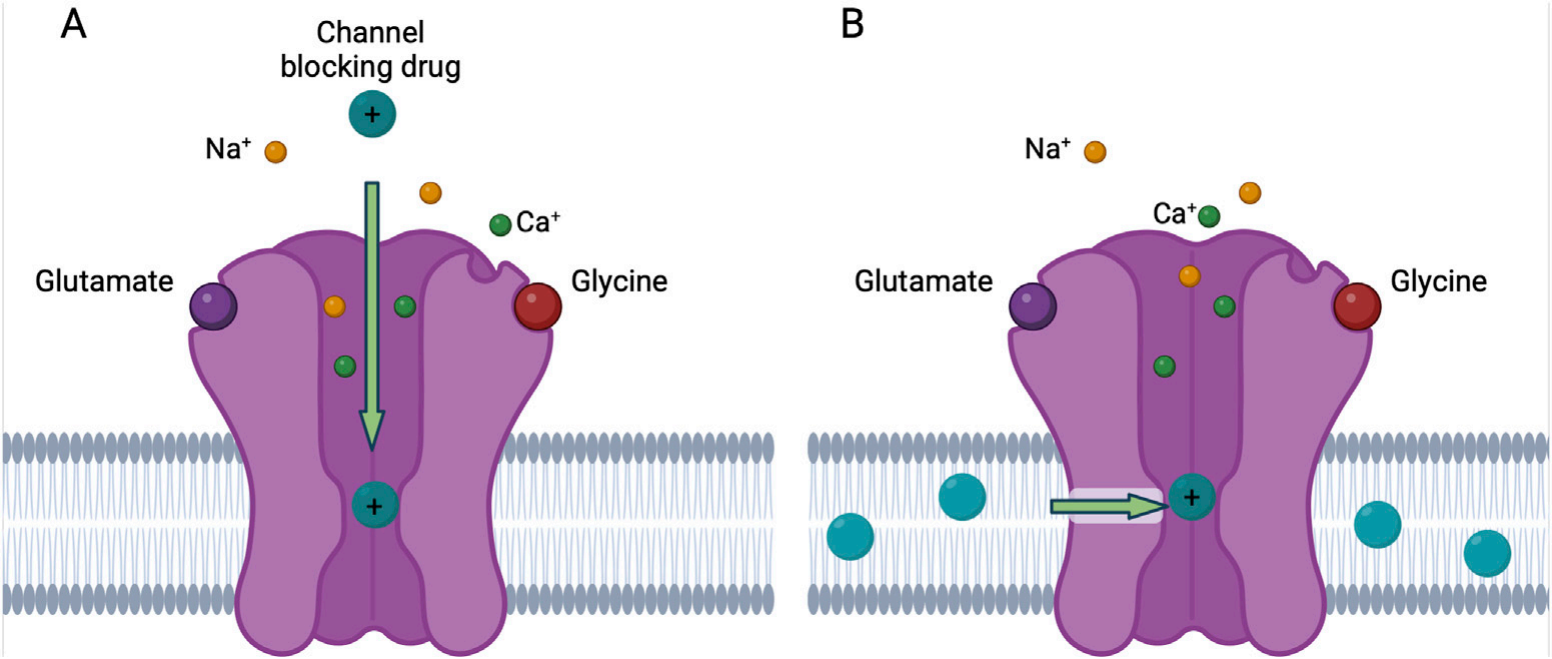
Traditional block and MCI of NMDARs by channel blocking drug. **(A)** In traditional block, charged molecules of the channel blocking drug (teal) enter the open NMDAR channel from the extracellular solution and bind to the deep site. **(B)** In membrane to channel inhibition (MCI), uncharged molecules of channel blocking drug enter the plasma membrane and then traverse a fenestration (green arrow) when the NMDAR is in the open conformation and bind to the deep site. The uncharged molecule of channel blocking drug gains a charged hydrogen ion when bound to the deep site. Figure created with BioRender.

There are two known routes through which NMDAR channel blocking drugs can access their binding site (the “deep site”) near the N-site at the tips of the M2 re-entrant loops (Kashiwagi et al., 2002; Chen and Lipton, 2005; Phillips et al., 2020). The most well studied route is “traditional” channel block, which occurs when a channel blocking drug enters the open NMDAR channel directly from the extracellular solution (Figure 2A). For many years, this was the only known route of access to the deep site for channel blocking drugs. However, in 2022 a second route of entry to the deep site was described (Wilcox et al., 2022). Inhibition via this second route, called membrane to channel inhibition (MCI), involves access of channel blocking drug to the deep site from within the plasma membrane. In MCI, uncharged molecules of channel blocking drug first enter the plasma membrane. Receptor activation then allows transit of blocker molecules through a gated, lateral fenestration in the NMDAR into the deep site (Figure 2B). MCI is exhibited by most tested NMDAR channel blocking drugs, including memantine, MK-801, PCP, and dextrorphan (Wilcox et al., 2022). The notable exception is ketamine, which is the only NMDAR channel blocking drug we have tested so far that does not appear to exhibit MCI (Kotermanski et al., 2009).

Prior to the identification of MCI, there was extensive evidence for a mechanism of inhibition by memantine in addition to traditional block (Blanpied et al., 1997; Sobolevsky and Koshelev, 1998; Sobolevsky, 1999; Bolshakov et al., 2003; Chen and Lipton, 2005; Kotermanski et al., 2009; Glasgow et al., 2018). This second form of inhibition was initially hypothesized to result from drug binding to a site on NMDARs other than the deep site, and was referred to as second site inhibition (SSI) (Kotermanski et al., 2009; Glasgow et al., 2018). Further experiments revealed that the phenomenon referred to as SSI did not involve a second inhibitory site; inhibition still resulted from blocker occupancy of the deep site (Wilcox et al., 2022). SSI therefore was renamed MCI. Note that channel blockers may act via yet additional inhibitory mechanisms. For example, a form of NMDAR inhibition similar to MCI was observed 10 s after elimination of memantine from the extracellular solution (Sobolevsky et al., 1998), a time course inconsistent with the kinetics of memantine exit from the membrane during MCI (see Section 2.3).

Although the same channel blocking site is involved in both traditional block and MCI, drugs may have different attributes when accessing the deep site through distinct paths. For example, all tested channel blocking drugs have much lower affinity when acting through MCI than through traditional block. Memantine has an IC_50_ of approximately 1.5 μM at −65 mV when acting through traditional block on GluN1/2A receptors expressed in tsA201 cells (Glasgow et al., 2017); similar values have been observed in a variety of other preparations (Chen et al., 1992; Parsons et al., 1995; Blanpied et al., 1997; Sobolevsky et al., 1998). However, memantine has an IC_50_ of 71 μM at −65 mV when acting through MCI on GluN1/2A receptors expressed in tsA201 cells (Wilcox et al., 2022). The small amount of uncharged channel blocking drug typically present in physiological solution likely contributes to memantine’s lower affinity when acting through MCI. At physiological pH, all drugs tested thus far exist primarily in a charged state, and entry of charged drug molecules into the membrane is energetically unfavorable. For example, at a pH of 7.2, only about 0.06% of memantine is uncharged due to its pKa of approximately 10.4. Thus, if 100 μM memantine is in aqueous solution at a pH of 7.2, the aqueous concentration of uncharged memantine is approximately 60 nM. Memantine MCI nevertheless reduces NMDAR mediated current by approximately 40% under these conditions because uncharged memantine, which is very hydrophobic, accumulates at vastly higher concentrations within the membrane (Wilcox et al., 2022). Hypothetically, a drug that is predominantly uncharged in aqueous solution and therefore preferentially resides in the plasma membrane may inhibit with greater potency through MCI than through traditional block.

### 2.3 Isolating MCI experimentally

**FIGURE 3:**
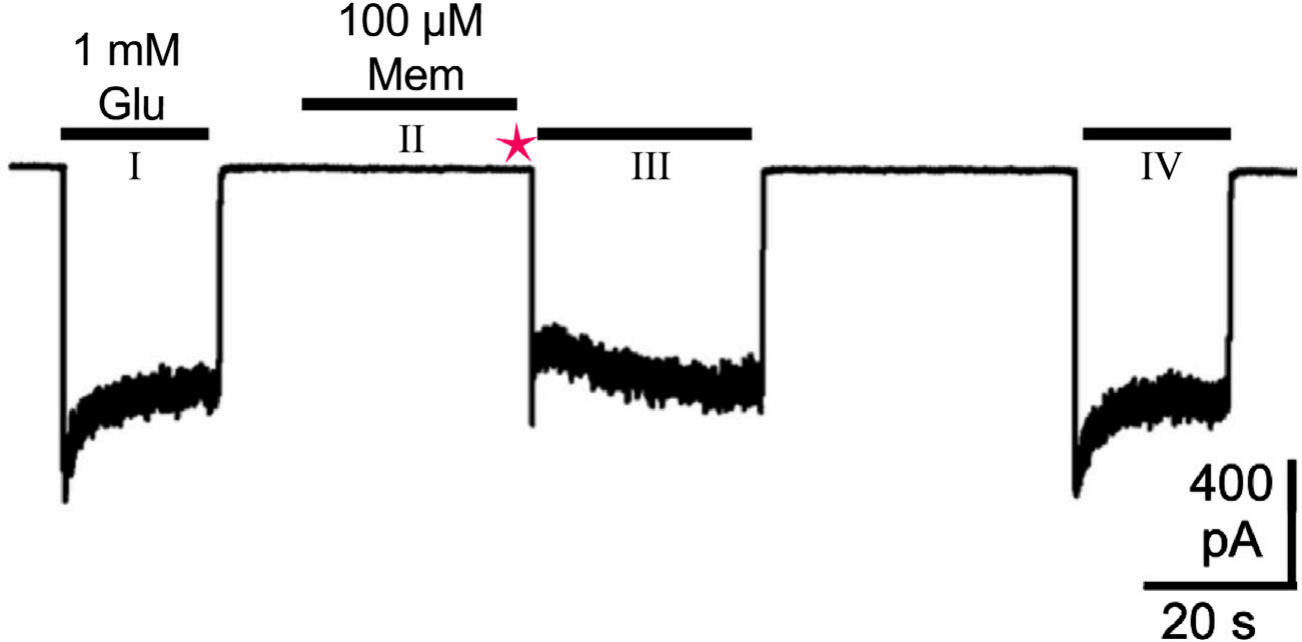
Protocol for study of MCI, modified from Figure 1A in Wilcox et al. (2022). A whole-cell patch-clamp recording at −65 mV from a tsA201 cell transfected to express GluN1/2A receptors is shown. The first (I) and last (IV) 1 mM glutamate (Glu) applications were performed to activate control responses, without pre-exposure to memantine (Mem). The middle application of 1 mM Glu (III) was used to quantify MCI. The middle 1 mM Glu application (III) was preceded by: a 30-s application of 100 μM Mem in the absence of agonist (II), during which Mem entered the cell’s plasma membrane but not the NMDAR; a 1-s wash with control solution (magenta star), which washed away extracellular Mem to prevent subsequent inhibition via traditional block. The observed transient inhibition during the middle 1 mM Glu application (III) therefore resulted from membrane-associated Mem acting through MCI, and not from traditional block, since no extracellular Mem remains during the middle Glu application (III). MCI was quantified by normalizing the current following the middle 1 mM Glu application (III) to the average of the two control responses resulting from the first (I) and last (IV) 1 mM Glu applications.

Technical considerations shape our current understanding of MCI. Wilcox et al. (2022) examined MCI using whole-cell patch clamp electrophysiology applied to cultured neurons and to tsA201 cells transfected to express NMDARs. To characterize MCI without interference from inhibition via traditional block, the following protocol was used (Figure 3): First, agonist (glutamate without channel blocking drug; glycine was present in all solutions) was applied extracellularly to the cell under study to allow measurement of the control NMDAR-mediated current. Then, in the absence of agonist, NMDAR channel blocking drug was applied. When applied alone, uncharged channel blocking drug can access the plasma membrane but not the NMDAR channel since receptor activation is required for channel blocking drug to bind to the deep site. Then, all channel blocking drug in the extracellular solution was eliminated by washing the cell with drug-free solution using a fast perfusion system (Glasgow et al., 2017). The solution exchange time constant of this system is <30 ms, ensuring that all extracellular drug was eliminated within the 1-s wash. Agonist without channel blocking drug was then applied, resulting in NMDAR activation. An immediate inward current was observed before channel blocking drug could transit from the membrane through the fenestration to the deep site. The presence of this initial current indicates that, unlike VGSC blocking drugs (see Section 3.1), NMDAR channel blocking drugs cannot access the channel until the receptor is activated. When the fenestration is opened as a result of NMDAR activation, channel blocking drug can transit to its blocking site, causing a rapid decay of current after the initial peak. This peak current following channel blocking drug application and washout is slightly smaller than the peak control current because of the slow activation kinetics of NMDARs (Erreger et al., 2005), allowing channel blocking drug to act on early-opening NMDARs. The inward current then recovered to the same steady-state level as observed during the control glutamate application as channel blocking drug both exited the membrane and unblocked from the NMDARs. The agonist application that followed application of channel blocking drug and the 1-s wash was long enough to allow complete recovery from channel block. Finally, another control application of glutamate without pre-exposure to channel blocking drug was performed. MCI was quantified by comparing current amplitude during the glutamate application made 1 s after application of channel blocking drug to the average of the control currents measured before and after MCI induction.

While this approach is effective in isolating MCI from traditional block, it is not without limitations. First, the 1-s wash partially obscures the full effect of MCI. The duration of the wash step is sufficient to eliminate extracellular drug, as is essential for isolating MCI from traditional block. However, while this wash is occurring, memantine is continuously diffusing out of the membrane with a time constant of approximately 2 s (Kotermanski et al., 2009). Thus, the amount of membrane-associated drug is reduced by the time the wash ends and MCI is measured. Second, given the essentiality of the wash, studying MCI in a brain slice is challenging because rapid and complete exchange of the extracellular solution in slices is not feasible.

It may be possible to circumvent these issues by using other techniques to investigate MCI. For example, intracellular application of channel blocking drug may act through MCI (Wilcox et al., 2022), a hypothesis being tested in ongoing experiments (Neureiter et al., 2023). The mechanism of action underlying NMDAR inhibition by intracellular channel blocking drug is unknown, even though intracellular application of MK-801 is commonly used experimentally to inhibit NMDARs. It is unlikely that intracellular drug can access the deep site by traversing the selectivity filter directly from the cytoplasm because the constriction between the cytoplasm and the deep site is too narrow in NMDARs (Villarroel et al., 1995). Intracellular drug may instead act through MCI due to the accessibility of the plasma membrane from the intracellular solution. Using intracellular application of drug to study MCI would circumvent the limitations of the 1-s wash during the MCI protocol (Figure 3). Therefore, intracellular application of channel blocking drug may offer a way to isolate and study MCI in addition to extracellular application of channel blocking drug.

### 2.4 Ketamine and MCI

It is unknown why ketamine does not appear to exhibit MCI. Even 500 µM ketamine, about 1,000 times greater than the IC_50_ of ketamine for traditional block of GluN1/2A receptors, does not produce observable MCI (Kotermanski et al., 2009). Ketamine has a lower pKa than memantine (7.5 versus 10.4), meaning that at equivalent total aqueous concentrations, there is a higher concentration of uncharged ketamine than uncharged memantine in aqueous solution. Since uncharged drug is much more likely to enter the plasma membrane, a drug with lower pKa should hypothetically cause stronger MCI given similar affinities and ability to traverse the fenestration. One hypothesis is that ketamine cannot transit through the fenestration due to unfavorable interactions with fenestration-lining amino acid residues, preventing it from accessing the deep site from the plasma membrane. Given that the protocol for measuring MCI currently depends on a 1-s wash (see Section 2.3), an alternative hypothesis is that the rate of ketamine exit from the membrane is so fast that MCI is not measurable using established protocols. Further experiments are needed to investigate these possibilities.

### 2.5 Drug-membrane interaction

An important feature of MCI that distinguishes it from traditional block is the interaction of channel blocking drug with the plasma membrane prior to interaction with the NMDAR channel itself. The plasma membrane is a heterogeneous and complex structure composed of many different proteins and lipids. In addition to separating the intracellular and extracellular compartments, the plasma membrane is responsible for a wide variety of structural and signaling functions (Casares et al., 2019). Lipid rafts are microdomains in the plasma membrane that are enriched with cholesterol and sphingolipids and are particularly important in protein trafficking and cell signaling (Brown and London, 1998; Simons and Toomre, 2000; Ikonen, 2001). It is unknown how lipid composition may affect the rate of drug entry into or diffusion through the plasma membrane and therefore MCI. Experimental manipulation of NMDAR-lipid raft colocalization and overall membrane density and composition could be used to shed light on the effect of membrane properties on MCI. Potentially useful experimental approaches include knockout of the scaffolding protein p140Cap to reduce NMDAR-lipid raft colocalization (Angelini et al., 2022), and membrane cholesterol depletion with methyl-beta-cyclodextrin or statins (Fassbender et al., 2001; Korinek et al., 2020). MCI is dependent on the amount of NMDAR channel blocking drug within the membrane; however, very little is known about the kinetics of drug entry to and exit from the plasma membrane. Membrane capacitance measurements might provide a way to infer the rate of drug association and exit from the membrane (Lindau and Neher, 1988; Vyklicky et al., 2015). These approaches may be useful for exploring the impact of membrane composition on MCI.

### 2.6 Neurosteroids and MCI

Membrane-associated neurosteroids may also influence MCI. Membrane cholesterol is essential for NMDAR function (Korinek et al., 2020), and is required for the synthesis of neurosteroids, which modulate neuronal excitability partially through action on NMDARs (Vyklicky et al., 2014; Blanco et al., 2018). The amino acid asparagine (N) at position 650 on the GluN1 subunit is involved in mediating the effects of the neurosteroid 24(S)-hydroxysterol (24(S)) on NMDARs (Tang et al., 2023). Another study found that same residue, when mutated to a lysine (GluN1(N650K)), affected MCI by memantine (Kolcheva et al., 2023). However, it is unknown whether 24(S), or any other neurosteroid, can directly affect MCI. As neuromodulators, neurosteroids likely influence MCI as a result of modulating overall NMDAR function; it may also be possible for the presence of modulatory factors within the membrane to have a direct physical influence on a drug’s ability to enter the fenestration, although this idea has not been tested.

### 2.7 Future directions

A wide range of questions concerning the properties and implications of NMDAR MCI remain unanswered. Topics of interest that we hope to address in the near future include: whether NMDAR channel blocking drugs exhibit MCI-specific NMDAR subtype dependence; whether channel blocking drugs exhibit weaker voltage dependence when acting through MCI than when acting through traditional block because the uncharged form of the drug binds in the channel (Nigam et al., 2023; Neureiter et al., 2024); whether intracellular channel blocking drugs act through MCI (Neureiter et al., 2024). Should characteristics such as NMDAR subtype specificity or voltage dependence differ between MCI and traditional block, these features may be harnessed for clinical and experimental applications by development of drugs that inhibit preferentially through MCI (Misiachna et al., 2024). In addition, investigation of MCI has been mostly limited to experiments with memantine and GluN1/2A receptors; much remains to be learned about MCI by other drugs and in other NMDAR subtypes.

MCI is an exciting, recently discovered mechanism of NMDAR inhibition by channel blocking drugs. However, membrane associated mechanisms of channel block are known to exist for other types of channels. In fact, the first investigation of MCI was influenced by the experiments of Bertil Hille and colleagues (Hille, 2001) in their study of inhibition of voltage-gated sodium channels by local anesthetics.

## 3 Introduction to VGSCs

Voltage-gated sodium channels (VGSCs) are one of the most prominent ion channel types known to be inhibited by drugs that act via a hydrophobic path. VGSCs are pore-forming transmembrane proteins that conduct Na^+^ current in a voltage-dependent manner. Widely expressed in the membranes of neurons, myocytes, and other electrically excitable cells (Hodgkin and Huxley, 1952; Catterall, 2000), VGSCs are crucial signaling proteins responsible for the initiation and propagation of the action potential (Hodgkin and Huxley, 1952; Hille, 2001). VGSCs also are expressed at lower levels in non-excitable cells, where they play diverse roles (Black and Waxman, 2013).

Structurally, VGSCs are composed of a single α subunit that can associate with one or more smaller auxiliary β subunits (Catterall, 2000). The ion-conducting pore and the voltage sensors are formed by the α subunit, as demonstrated by heterologous expression experiments wherein expression of the α subunit alone is sufficient to produce the essential aspects of VGSC function (i.e., voltage-dependent channel gating, ion selectivity, rapid inactivation). β subunit expression, though not required, modulates key properties such as voltage dependence, gating, kinetics, and localization (Bouza and Isom, 2018). Although α subunits Na_V_1.1-Na_V_1.9 have strong sequence homology, each subunit has unique functional properties and expression patterns (Noreng et al., 2021). A 10th related α subunit isoform, NaX, also exists, though it does not function as a voltage-gated channel (Goldin, 2002; Dolivo et al., 2021).

The VGSC α subunit is predicted to fold into a pseudo-tetrameric structure composed of four similar domains (I-IV), each containing six α-helical transmembrane segments (S1-S6) and a re-entrant loop between S5 and S6. The S4 segment of each domain serves as the voltage sensor due to positively charged amino-acid residues in every third position on the segment. Small depolarizations of the transmembrane potential result in S4 segments sliding upwards towards the extracellular solution, altering channel conformation and allowing Na^+^ ions to traverse the pore (Martin et al., 2020). The extracellular portion of the pore is formed by re-entrant loops between the S5 and S6 helices. These loops form the narrowest region of the pore and serve as the selectivity filter. The intracellular gate of the pore is formed by the four S6 segments.

VGSC channelopathies and resulting dysregulation of Na^+^ flux have been implicated in many disorders, including epilepsy, chronic pain, cardiac arrhythmia, ataxia, migraine, and autism spectrum disorder (Lampert et al., 2010; Mantegazza et al., 2021; Meisler et al., 2021; Indelicato and Boesch, 2023). Treating pathologies that result from VGSC dysfunction is difficult; because of their near ubiquitous role in neurotransmission, effective therapeutics cannot simply globally inhibit VGSC activity.

### 3.1 VGSC blocking drugs

Local anesthetics (LAs), antiarrhythmics (ARs), and antiepileptics (AEs) are clinically important drugs that target VGSCs and bind within the VGSC central cavity (Ahern et al., 2008; Pless et al., 2011). These drugs inhibit Na^+^ currents via at least two mechanisms: by obstructing current flow as a result of steric hindrance or electrostatic interactions with Na^+^, and by modifying VGSC gating kinetics (Hille, 1977a; Hondeghem and Katzung, 1984; Catterall, 1987; Hille, 2001). X-ray crystallographic structures of a bacterial voltage-gated sodium channel (NaVAb) in complex with the LA lidocaine and the AR flecainide (Payandeh et al., 2011; Gamal El-Din and Lenaeus, 2011) confirmed that these drugs can inhibit Na^+^ flux by physically blocking the channel along the pore-lining side of the S6 segments (Figure 1D) (Ragsdale et al., 1994; Yarov-Yarovoy et al., 2002). The electron density for lidocaine bound to the VGSC was found in the central cavity on the intracellular side of the selectivity filter (Gamal El-Din et al., 2018), ideally situated for impeding ion permeation. Flecainide, while much larger than lidocaine, took on a very similar binding position within the central cavity (Payandeh et al., 2011; Gamal El-Din et al., 2018). In addition to physically obstructing the channel pore, many LAs, ARs, and AEs also inhibit VGSCs by modifying channel gating kinetics and voltage dependence as they preferentially bind to and stabilize VGSCs in nonconducting inactivated states. The net effect of both pore obstruction and gating modification is a decrease in a neuron’s VGSC-mediated Na^+^ current and action potential frequency.

Similar to NMDAR channel blocking drugs, many VGSC channel blocking drugs have both charged and uncharged forms. As addressed below, the charge of a drug directly influences its ability to use hydrophobic or hydrophilic paths to access its site of action.

### 3.2 Modulated receptor hypothesis

Several models have been proposed to explain the complex mechanisms of inhibition by VGSC blockers. The Modulated Receptor Hypothesis (MRH), originally proposed as a model of the mechanism of action of LAs on VGSCs (Hille, 1977a), remains among the most prominent. The MRH is based on data that suggest the binding affinity of LAs varies as a function of VGSC gating state: open and inactivated channel states exhibit higher drug affinities than the closed state. There is evidence supporting state-dependent binding by many LAs. Lidocaine, for example, binds to wild-type Na_V_1.5 VGSCs with an IC_50_ of approximately 36 μM, whereas the apparent K_D_ (estimated from binding kinetics) for mutant channels incapable of inactivation was approximately 600 μM. Closed channels exhibit significantly lower drug affinity (Bennett et al., 1995). To explain how LAs can directly bind VGSCs in the closed and inactivated state, the MRH predicted the existence of hydrophobic pathways that permit drug access to the central cavity (Hille, 1977a). 34 years later, hydrophobic pathways fitting this description were identified within the X-ray crystallographic structure of NavAb in a closed-pore conformation; the solved structure revealed four symmetric lateral fenestrations within the walls of the central cavity (Payandeh et al., 2011; Noreng et al., 2021).

The MRH does not fully explain the intricacies of LA antagonism of VGSCs. For example, the MRH operates on the assumption that there is a single LA binding site in the VGSC central cavity, whereas more recent experiments suggest there may be separate binding sites for charged versus neutral forms of certain drugs (Buyan et al., 2018). Hybrid models that incorporate ideas from the MRH and other models, such as the Guarded Receptor Hypothesis (Starmer et al., 1984), are likely needed to fully encapsulate mechanisms of VGSC inhibition (O'Leary and Chahine, 2018).

### 3.3 Fenestrations in VGSCs

Several years after their discovery in NavAb channels, lateral fenestrations in the VGSC transmembrane domain (TMD) were also identified in eukaryotic orthologs (Shen et al., 2017; Yan et al., 2017; Pan et al., 2018). Many hydrophobic VGSC blockers can use these fenestrations to access the central cavity binding sites directly from the lipid bilayer (Payandeh et al., 2011; Gamal El-Din et al., 2018). Crystal structures suggest that these fenestrations are drug-accessible even in nonconducting VGSC states (Payandeh et al., 2011), in contrast to the NMDAR fenestration that appears to permit MCI only when the channel is open. Mutations of fenestration-lining residues to constrict or enlarge the fenestration radius show graded effects on slow resting-state block by flecainide, lidocaine, and benzocaine (Gamal El-Din et al., 2018). These studies inspired experiments with NMDARs to examine the involvement of residue GluN2A (M630) in the fenestration used by the channel blocker memantine to access the deep site (Wilcox et al., 2022).

### 3.4 Hydrophilic and hydrophobic paths to inhibitory sites in VGSCs

Depending on drug properties such as molecular size, functional group identity, and amphiphilicity, VGSC blockers can use hydrophilic and/or hydrophobic paths to reach their central pore binding sites and inhibit Na^+^ flux (Hille, 1977b; Courtney, 1980). Charged VGSC blockers typically use hydrophilic routes to access their binding site in the VGSC central pore, as it is energetically unfavorable for charged molecules to enter the hydrophobic plasma membrane. The primary hydrophilic path involves charged drug traveling from the cytoplasm through the intracellular VGSC activation gate to access the central pore. Most evidence suggests that this hydrophilic path is only accessible when the VGSC activation gate is open ((Strichartz, 1973; Hille, 1977a; Starmer et al., 1984; Payandeh et al., 2011) but see (Boiteux et al., 2014)). Thus, compounds that rely on the hydrophilic pathway tend to bind only when the VGSC is in the open state. Permanently charged quaternary LAs have minimal effect when applied extracellularly, suggesting they are incapable of accessing their central pore binding site directly from the extracellular solution (Strichartz, 1973). Some hydrophilic VGSC blockers such as tetrodotoxin and μ-conotoxin do act by binding at the extracellular mouth of the pore external to the selectivity filter (Pan et al., 2019; Shen et al., 2019), but this is not a primary pathway of inhibition for clinically relevant hydrophilic VGSC blockers (Hille, 1977a). In contrast, uncharged VGSC blockers readily enter the hydrophobic plasma membrane and access their binding sites in the central pore via lateral fenestrations in the VGSC TMD (see Section 3.3). Most LAs, ARs, and AEs are secondary or tertiary amines, which in aqueous solution exist in positively charged and uncharged forms in an equilibrium dependent on drug pKa and solution pH. Drugs of this nature can utilize both hydrophobic and hydrophilic paths to access VGSC central pore binding sites (Frazier et al., 1970; Strichartz, 1973; Hille, 1977b).

To better understand the relative effectiveness of charged versus uncharged VGSC blockers, experiments were performed to evaluate how drug potency depends on pH. These experiments led to the conclusion that tertiary amine LAs are most potent when applied intracellularly at an acidic pH (Narahashi et al., 1970), and more broadly that they are more potent in their protonated form (Nettleton and Wang, 1990). This suggests that for VGSC blockers that exist in both charged and uncharged forms at physiological pH, entry of the positively charged form of drug into the central pore directly from the cytoplasm is the most potent inhibitory pathway.

Recovery of VGSCs from block by extracellularly applied secondary or tertiary amine LAs is slowed by a more acidic extracellular pH, whereas intracellular pH has no effect (Schwarz et al., 1977; Grant et al., 1980; Bean et al., 1983). These experiments have suggested that extracellular protons may permeate the VGSC pore and associate with bound, uncharged drug, thus preventing its escape via hydrophobic lateral fenestration pathways. Further support for this idea is based on two findings from experiments with permanently charged quaternary ammonium homologues of LAs. First, permanently charged LA homologues are more potent when applied intracellularly than when applied extracellularly; second, their potency is unaffected by changes in intracellular or extracellular pH (Frazier et al., 1970).

### 3.5 *In-silico* insights into VGSC blocker mechanisms

Findings demonstrating more effective VGSC inhibition from the intracellular solution by protonated rather than by uncharged channel blocking drug forms may seem surprising, since their binding sites in VGSCs are lined by hydrophobic residues. Molecular Dynamics simulations have suggested that the VGSC pore may contain distinct binding sites for uncharged versus charged drug (Buyan et al., 2018). These simulations identified, in addition to the well-defined antagonist binding site on the pore-lining side of the S6 segments (thought to be preferred by uncharged drug), another slightly more extracellular site within the central cavity. Binding of charged blockers to the more extracellular site is energetically favorable because the drug’s positive charge orients toward negatively charged selectivity filter residues.

Further complexity is suggested by simulations that show the LAs benzocaine and lidocaine accessing fenestrations from the extracellular solution by traversing a hydrophobic path through the VGSC without diffusing into the membrane (Boiteux et al., 2014; Nguyen et al., 2019). In this alternative hydrophobic path to the fenestrations, LAs maintain contact with the VGSC surface. Thus it has been suggested that hydrophobic VGSC residues along this path act as transient binding sites (Martin and Corry, 2014). These data suggest that hydrophobic VGSC blockers may not need to diffuse into the plasma membrane to access fenestrations to their central cavity binding site. There is also physiological support for the alternative hydrophobic path through the VGSC: voltage-clamp recordings from VGSCs with mutations that introduce small, polar residues along the hypothesized hydrophobic path allow quaternary homologs of lidocaine to utilize this non-membranous path despite their permanent positive charge (Qu et al., 1995; Sunami et al., 2000).

### 3.6 Negative allosteric modulation of VGSCs by membrane-associated drugs

Many other membrane-associated drugs act as negative allosteric modulators of VGSCs (Carnevale and Klein, 2017; Li et al., 2024). An example is cannabidiol (CBD), a major nonpsychoactive component of cannabis that is FDA-approved to treat certain epilepsies (Devinsky et al., 2019; Thiele et al., 2019), and also is used to relieve pain (Xu et al., 2020; Alaia et al., 2022). CBD interacts with a vast array of intracellular and membrane proteins in addition to VGSCs. However, CBD at sub-micromolar concentrations exhibits substantial effects at only a few of these targets (Huang et al., 2023). Among the proteins inhibited by low-dose CBD are Na_V_1.7 (Huang et al., 2023) and Na_V_1.8 (Zhang and Bean, 2021), VGSC subtypes that play essential roles in pathological pain (Hameed, 2019). CBD is extremely lipophilic; with a logP of 6.33, CBD is approximately 10^6^ times more concentrated in the lipid membrane compared to the aqueous intracellular or extracellular solutions (Huang et al., 2023). Cryo-EM structures of CBD bound to Na_V_1.7 suggest two different binding sites, one of which is in the fenestration between domains IV and I (Huang et al., 2023). CBD is a negative allosteric modulator of VGSCs and not a channel blocker, as neither of CBD’s Na_V_1.7 binding sites suggest occlusion of the channel pore. Based on its lipophilicity CBD likely accesses its binding sites directly from the lipid membrane. Therefore, VGSCs can be inhibited by membrane-associated drugs with a range of mechanisms of action.

## 4 Modulation of other ion channels by membrane-associated drugs

We have thus far explored the relatively well-established actions of membrane-associated drugs that inhibit NMDARs and VGSCs. There is also strong evidence that similar mechanisms are involved in inhibition of other channel types, including K_V_ channels, VGCCs, AMPARs, KARs, nAChRs, GABA_A_Rs, and TRP channels. We will explore the relevant literature in the following sections.

### 4.1 KV channels

Voltage-gated potassium (KV) channels are a strongly evolutionarily conserved class of ion channels (Hille, 2001) found across a wide variety of species and in many cell types. KV channels are encoded by 40 genes and are broadly grouped into 12 families (Grizel et al., 2014; Ranjan et al., 2019). KV channels are tetramers composed of four α subunits with optional auxiliary β subunits (Abbott, 2022), allowing diverse functionality. Each α subunit has six transmembrane segments, S1-S6, and a re-entrant loop between S5 and S6. The pore region is formed by S5, S6, and the re-entrant loop, and the S4 segment serves as the voltage sensor (Grizel et al., 2014). KV channels allow K^+^ to permeate with remarkable selectivity in response to changes in membrane potential (Hille, 2001). Most types of KV channels inactivate via slow (C-type) and fast (N-type, or ball-and-chain) inactivation (Kim and Nimigean, 2016), the combination of which allows for tight regulation of ion flow, which is extremely important in neural transmission.

Drugs belonging to the broad category of K_V_ channel antagonists are used for a wide variety of clinical applications, particularly to treat diseases related to cellular excitability, and for experimental applications. Some K_V_ channel antagonists have shown evidence of interaction with hydrophobic sites on K_V_ channels. For example, gambierol inhibits K_V_1 and K_V_3 channels by interacting with residues on the S6 transmembrane helix and modifying gating characteristics (Kopljar et al., 2009). Gambierol is very likely to bind K_V_ channels from the membrane based on its strong lipophilicity and ability to interact with closed K_V_ channels from either side of the membrane. Psora-4 is a K_V_3.1 and K_V_1.5 channel blocking antagonist that also binds in a lipophilic pocket, stabilizing a nonconducting channel state (Marzian et al., 2013). Many other compounds are known to affect K_V_ channels through interactions with hydrophobic binding sites, including retigabine, polyunsaturated fatty acids (PUFAs), and derivatives of dehydroabietic acid (Lange et al., 2009; Borjesson and Elinder, 2011; Ottosson et al., 2017; Van Theemsche et al., 2020).

The pharmacology of hERG channels (K_V_11.1) has been particularly well investigated due to the channels’ involvement in long QT syndrome (Vandenberg et al., 2012; Chiamvimonvat et al., 2017). hERG channels are modulable by a wide range of endogenous and synthetic compounds, some of which access their binding site through hydrophobic paths (Kudaibergenova et al., 2019). Ivabradine is FDA-approved to treat angina and is used in patients with an intolerance to beta blockers. Ivabradine inhibits hERG channels at clinically relevant concentrations in a state dependent manner, in addition to acting on HCN channels as a major target (Lees-Miller et al., 2015; Melgari et al., 2015). Molecular dynamics simulations have shown that ivabradine may use a hydrophobic route to access its binding site within hERG channels (Lees-Miller et al., 2015).

Adamantane derivatives were used to identify a fenestration in K_V_7.1 channels that is accessible only when a KCNE1 accessory subunit was bound (Wrobel et al., 2016), broadly reminiscent of the state dependence of MCI of NMDARs. Other types of potassium channels also contain fenestrations relevant to drug action (Jorgensen et al., 2016); structural homology between potassium channel types may help in the identification of similar pathways in K_V_ channels.

### 4.2 VGCCs

Voltage-gated calcium channels (VGCCs) are voltage-dependent ion channels that are selectively permeable to Ca^2+^ ions. Entry of Ca^2+^ into cells through VGCCs regulates many processes within and outside the nervous system, including neurotransmitter release, activation of other channels, activation of second messenger pathways, and muscle contraction (Hille, 2001).

Functional VGCCs (like VGSCs) require only a single α subunit; however, biochemically isolated VGCCs have been found to contain additional regulatory subunits. There are many VGCC subunits, including the α_1A_ -α_1I_, α_1S,_ α_2_, β_1-4_, γ_1-8_, and δ_1_ types (Ertel et al., 2000; Buraei and Yang, 2010; Loh et al., 2023). The structure of the α_1_ subunit is also topologically similar to the VGSC α subunit, consisting of four domains (I-IV), each with six α-helical transmembrane segments (S1-S6) and a re-entrant loop between S5 and S6. The S4 segment serves as the voltage sensor.

Similarities in the amino acid sequences of α subunit types can be used to classify VGCCs. The Ca_V_1 family includes channels containing α_1S_ (Ca_V_1.1), α_1C_ (Ca_V_1.2), α_1D_ (Ca_V_1.3), and α_1F_ (Ca_V_1.4) subunits; the Ca_V_2 family includes channels containing α_1A_ (Ca_V_2.1), α_1B_ (Ca_V_2.2), and α_1E_ (Ca_V_2.3) subunits; the Ca_V_3 family includes channels containing α_1G_ (Ca_V_3.1), α_1H_ (Ca_V_3.2), and α_1I_ (Ca_V_3.3) subunits. Each distinct VGCC family also mediates Ca^2+^ currents with different specific characteristics including activation and deactivation kinetics. The Ca_V_1 channel family mediates L-type Ca^2+^ currents, the Ca_V_3 channel family mediates T-type Ca^2+^ currents, and Ca_V_2.1, Ca_V_2.2, and Ca_V_2.3 channels mediate P/Q-type, N-type, and R-type Ca^2+^ currents, respectively (Nowycky et al., 1985; Ertel et al., 2000; Wu et al., 2016). VGCCs can also be classified based purely on their voltage dependence. Low voltage activated (LVA) channels (Ca_V_3 family) require only relatively small depolarizations to activate, while stronger depolarizations are required for high voltage activated (HVA) channels (Ca_V_1 and Ca_V_2 families) to activate (Hille, 2001).

VGCCs are extremely important physiologically and pathophysiologically due to their ubiquity and essential functions (Kessi et al., 2021). Therefore, channel block of VGCCs has been extensively studied (Godfraind, 2017). There are many FDA-approved VGCC antagonists that are used to treat diseases such as hypertension, chronic angina, and coronary heart disease (Elliott and Ram, 2011). Two major classes of clinically relevant VGCC inhibiting drugs are dihydropyridines, most of which act as allosteric inhibitors, and non-dihydropyridines, which act as channel blockers (Tang et al., 2016). Non-dihydropyridines can be further divided into phenylalkylamines and benzothiazepines based on their chemical structures (McKeever et al., 2024).

Dihydropyridines allosterically alter the conformation of the selectivity filter by binding within a fenestration at the interface of domains III and IV (Zhao et al., 2019a; Gao and Yan, 2021). There is also a fenestration in the Ca_V_3 channel at the interface between domains II and III, into which the small molecule Z944, a highly Ca_V_3 channel-selective blocker in phase II clinical trials for the treatment of seizures and neuropathic pain, can insert (Zhao et al., 2019b). In addition, the Ca_V_2 agonist GV-58 and its related analogs may bind to hydrophobic pockets or partition into the membrane and transit through a fenestration before accessing their binding sites (Wu et al., 2018). It is possible that VGCC blocking drugs, including non-dihydropyridines, access the channel pore via similar fenestrations, as has been shown in VGSCs and NMDARs. Ongoing research into the structural dynamics of VGCCs and exploration of hydrophobic paths to sites of inhibition by channel blocking drugs will enhance our ability to develop targeted therapies that can more effectively regulate VGCC activity.

### 4.3 AMPARs and KARs

α-amino-3-hydroxy-5-methyl-4-isoxazolepropionate receptors (AMPARs) and kainate receptors (KARs) are glutamatergic ion channels with strong structural homology to NMDARs (Figures 1A–C).

AMPARs are ionotropic glutamate receptors that mediate most fast synaptic transmission in the central nervous system. They are fundamentally involved in long term depression (LTD) and long term potentiation (LTP) (Diering and Huganir, 2018), and are implicated in many nervous system disorders including epilepsy and amyotrophic lateral sclerosis (Rogawski, 2011; Cull-Candy and Farrant, 2021). AMPARs are tetrameric complexes formed by GluA1-4 subunits as either homomers or heteromers, with each subunit imparting unique properties that are further tuned by auxiliary subunits (Kamalova and Nakagawa, 2021). Two broad categories of AMPARs exist: Ca^2+^ permeable AMPARs (CP-AMPARs) that lack the GluA2 subunit, and Ca^2+^ impermeable AMPARs (CI-AMPARs) that contain the GluA2 subunit. (Jonas et al., 1994; Brusa et al., 1995; Lalanne et al., 2018). Both CP-AMPARs and CI-AMPARs are permeable to Na^+^ and K^+^. CP-AMPARs have higher single channel conductance and play different roles in LTD and LTP than CI-AMPARs (Plant et al., 2006; Fortin et al., 2010; Sanderson et al., 2016). CP-AMPARs are also implicated in neurotoxicity (Cull-Candy and Farrant, 2021). Several AMPAR channel blocking antagonists have been found to be selective for CP-AMPARs, including IEM-1925, IEM-1460 and NASPM (Figure 1B) (Twomey et al., 2018).

While AMPAR channel blocking drugs typically enter the channel directly from the extracellular solution, Barygin et al. (2015) described a hydrophobic path of access for fluoxetine (Prozac). In addition to inhibiting AMPARs, fluoxetine is an SSRI and is frequently prescribed to treat a variety of psychiatric diseases, including MDD, obsessive-compulsive disorder, and panic disorders. Fluoxetine has higher affinity for CP-AMPARs than for CI-AMPARs, a feature that may be responsible for some of its clinical effects (Barygin et al., 2017). Uncharged molecules of fluoxetine can enter the plasma membrane (like in MCI) before occupying a site at the interface between two adjacent AMPAR subunits (Barygin et al., 2015). Although the precise path used by fluoxetine to access its binding site from the plasma membrane is unknown, structural investigations have demonstrated the existence of fenestrations within AMPARs (Herguedas et al., 2019). Other AMPAR antagonists that contain a titratable nitrogen, like desipramine, also have an uncharged form that may be able to access the plasma membrane before transiting to their site of action (Barygin et al., 2017). An active metabolite of fluoxetine, norfluoxetine, may also access its inhibitory site at the two-pore domain K^+^ channel TREK-2 through a hydrophobic path, binding to a state-dependent fenestration near the mouth of the cytoplasmic side of the receptor (Dong et al., 2015).

KARs, similar to AMPARs and NMDARs, are tetrameric receptors that are essential for neural signaling. KARs are commonly found at presynaptic as well as postsynaptic sites (Chalupnik and Szymanska, 2023). The GluK1-3 subunits (Collingridge et al., 2009) can form homomeric or heteromeric complexes, while GluK4 and GluK5 subunits are only able to form functional heteromers in combination with a GluK1, GluK2, or GluK3 subunit. Calcium permeability in KARs is imparted by the inclusion in the receptor of unedited GluK1 or GluK2 subunits (Lerma, 2003; Sun et al., 2009). Calcium permeable KARs (CP-KARs) can be blocked by a variety of drugs including NpTx-8, PhTx-74, Kukoamine A, and spermine (Figure 1C) (Gangwar et al., 2024). However, whether CP-KAR channel blocking drugs can access the channel from the membrane has not been explored. The potential for MCI-like mechanisms of KAR channel block is an interesting topic for future study.

### 4.4 nAChRs

Inhibitory drugs may also use a hydrophobic path to access their site of action in nicotinic acetylcholine receptors (nAChRs). nAChRs are ionotropic receptors that are found throughout the central and peripheral nervous systems. Muscle nAChRs mediate vertebrate muscle contraction (Hille, 2001). nAChRs conduct cations; some subtypes are highly permeable to Ca^2+^, which can mediate many of the effects of nAChR activation. nAChRs are pentameric receptors composed of α_1-10_, β_1-4_, γ, ε, and/or δ subunits, and can be either homomeric or heteromeric. The subunit composition of neuronal nAChRs, embryonic muscle nAChRs, and adult muscle nAChRs all differ (Itier and Bertrand, 2001). nAChR function is strongly implicated in addiction; receptor agonism by, for example, nicotine, leads to increased release of dopamine in the nucleus accumbens and initiation of addiction pathways (Besson et al., 2007; Leslie et al., 2013; Wittenberg et al., 2020). Dysfunction of nAChRs has been implicated in a wide variety of disease states including Alzheimer’s disease, addiction, Parkinson’s disease, Tourette’s syndrome, schizophrenia, and depression (Terry et al., 2023).

Many anesthetics bind to hydrophobic sites in nAChRs in addition to VGSCs. The LA lidocaine, which also inhibits VGSCs (see Section 3.4), has multiple inhibitory effects on nAChRs including the ability to bind to its channel blocking site while receptors are in the closed state (Alberola-Die et al., 2013). 2,6 dimethylamine (DMA), a molecule that closely resembles the hydrophobic moiety of lidocaine, was shown to associate with transmembrane, inter-subunit crevices in nAChRs (Alberola-Die et al., 2016). In addition, a variety of binding sites exist at the lipid-nAChR interface at which membrane phospholipids and uncharged LAs may compete (Mantipragada et al., 2003).

Memantine, which can act through MCI on NMDARs, can also block the channel of α7 nAChRs in rat hippocampal neurons and transfected *Xenopus* oocytes (Maskell et al., 2003; Aracava et al., 2005). It is possible that memantine can access its site of action on nAChRs through a hydrophobic path, as for NMDARs, although this possibility has not been directly tested. Such a pathway could be investigated using recently published high-resolution structures of α7 nAChRs (Delbart et al., 2018; Noviello et al., 2021; Burke et al., 2024). Given that nAChRs are extremely sensitive to their surrounding lipid environment (Sharp et al., 2019), a wide range of endogenous and synthetic steroids are likely to affect nAChRs through the plasma membrane (Barrantes et al., 2000).

### 4.5 GABA_A_Rs

γ-Aminobutyrate type A receptors (GABA_A_Rs) are ionotropic receptors that mediate inhibitory neurotransmission in the central nervous system through conduction of chloride ions across neuronal membranes (Ghit et al., 2021). GABA_A_Rs are heteropentamers, consisting of a combination of α_1-6_, β_1-3_, γ_1-3_, ρ_1-3_, δ, ε, π, and/or θ subunits. GABA_A_R dysfunction is involved in medical conditions such as epilepsy, autism, anxiety, and bipolar disorders (Ma et al., 2005; Collins et al., 2006; Macdonald et al., 2010; Ament et al., 2015; Nuss, 2015). GABA_A_Rs are targets of compounds including benzodiazepines, barbiturates, alcohols, and neurosteroids that can act either as positive or negative modulators (Ghit et al., 2021; Kim and Hibbs, 2021; Philip et al., 2024). Positive modulation of GABA_A_Rs is of particular interest in inducing a state of anesthesia, and in treating conditions such as anxiety and epilepsy. Thus, we will briefly consider here both positive and negative modulation of GABA_A_Rs by membrane-associated drugs.

Given the role played by GABA_A_Rs in inhibitory neurotransmission, nonspecific negative modulation of GABA_A_R function can promote conditions such as epilepsy, anxiety, and neurotoxicity (Ghit et al., 2021). However, negative allosteric modulators (NAMs) that act specifically on α5 GABA_A_Rs show promise in ameliorating cognitive impairment in conditions characterized by GABA_A_R overactivity (Jacob, 2019; Goeldner et al., 2022; Nuwer et al., 2023). GABA_A_R neurosteroid NAMs including dehydroepiandrosterone sulfate (DHEA-S), pregnenolone sulfate (PS), epipregnanolone, and isopregnanolone may be able to access their binding sites through the plasma membrane. DHEA-S acts at both the benzodiazepine binding site on the extracellular face of the receptor and at a second, lower affinity binding site, the exact location of which is unknown. DHEA-S may transit through the membrane to reach this second site, given that its binding depends on the properties of membrane lipids (Majewska et al., 1990; Demirgören et al., 1991). In addition, the endogenous neurosteroid 3α-hydroxy-5α-pregnan-20-one (APG) is a potent, clinically relevant (Balan et al., 2019) positive allosteric modulator of GABA_A_Rs (Majewska et al., 1986; Brinton, 1994) that binds to the GABA_A_R transmembrane α-β subunit interface (Sun et al., 2023).

Anesthetic compounds that potentiate GABA_A_Rs such as phenobarbital, etomidate, and propofol have binding sites within transmembrane interfacial cavities (Olsen, 2018; Kim et al., 2020; Kim and Hibbs, 2021). Specifically, phenobarbital was observed to bind at both the γ-β interface and the α-β interface (Kim et al., 2020). Interestingly, pentobarbital also inhibits AMPARs, on which its actions are voltage independent and pH dependent, features both consistent with a membrane-associated mechanism of action (Jackson et al., 2003). Barbiturate action is positively correlated with its lipid solubility, also consistent with a membrane-associated mechanism of action (Janoff et al., 1981).

### 4.6 TRP channels

Transient receptor potential (TRP) channels are ion channels involved in sensation of stimuli including heat, cold, pressure, pain, and light. TRP channels are tetramers, with each subunit containing six transmembrane helices (S1-S6) and a re-entrant loop between S5 and S6 (Zhang et al., 2023). The structure of TRP channels is very similar to that of voltage gated ion channels; however, positively charged residues are less densely grouped on TRP channel S4 segments than on the S4 segments of voltage-gated channels, imparting only weak voltage dependence to TRP channels (Cao, 2020). Although the majority of TRP channels are homotetramers, heterotetramers are also observed (Cheng et al., 2010). There are seven subfamilies of the mammalian TRP superfamily, consisting of TRPV (vanilloid), TRPA (ankyrin), TRPM (melastatin), TRPN (NO-mechanopotential), TRPC (canonical), TRPP (polycystin), and TRPML (mucolipin) (Zhang et al., 2023; Talyzina et al., 2025) type receptors, each with distinct roles in the signaling of sensation.

Fenestrations have been found in several members of the TRP channel superfamily. For example, a fenestration within TRPC3 is essential for gating modulation by the lipid messenger diacylglycerol (DAG) (Fan et al., 2018; Lichtenegger et al., 2018). The xanthine based TRPC1/4/5 inhibitor Pico145 has been hypothesized to integrate with the lipid membrane before interacting with its binding site, potentially through a fenestration as well (Wright et al., 2020). In addition, TRPM2 channels contain a fenestration connecting the cytosol to a large aqueous cavity within the channel, allowing Ca^2+^ to access its binding sites from the cytosol (Zhang et al., 2018; Szollosi, 2021). Fenestrations have also been found in TRPM and TRPP channels, although further investigation is needed to elucidate how they may be involved in the action of drugs or neurosteroids (Hulse et al., 2018; Yu et al., 2021).

The vanilloid site is a ligand binding site present in several types of TRP channels in the TMD between S1-S4 and the pore domains (Yelshanskaya and Sobolevsky, 2022; Talyzina et al., 2025). Several lipids including phosphatidyl-inositol, phosphatidyl-choline, and cholesterol hemisuccinate bind to the vanilloid binding site with a range of effects (Gao et al., 2016; Bhardwaj et al., 2020; Shimada et al., 2020; Zhang K. et al., 2021; Arnold et al., 2024; Rohacs, 2024). Derivatives of the small molecule (4-phenylcyclohexyl) piperazine (PCHPDs) are TRPV6 channel blocking drugs with additional weaker affinity for the vanilloid site (Bhardwaj et al., 2020); drugs that inhibit TRPV6 are of particularly strong clinical significance, given TRPV6’s overexpression in several types of cancer cells (Stewart, 2020). However, the paths used by modulatory compounds to access the vanilloid site, or other transmembrane binding sites, have not been elucidated.

## 5 Conclusion

Many types of drugs use hydrophobic paths to access their binding sites on a wide variety of essential channel types. The ability of certain drugs to associate with channels from within the plasma membrane may be related to their clinical effects (Miller, 2002; Forman et al., 2015). Herein, we have focused on the hydrophobic paths used by inhibitory drugs to access their binding sites in NMDARs, VGSCs, KV channels, VGCCs, AMPARs, KARs, nAChRs, GABA_A_Rs, and TRP channels. The limited characterization of NMDAR MCI that has been performed includes: identification of some channel blocking drugs that can act via MCI; pH dependence of MCI; demonstration that the membrane can act as a reservoir of uncharged drug molecules; and localization of a possible fenestration used by memantine (Wilcox et al., 2022). Other topics, including the properties of NMDAR MCI by channel blocking drugs beyond memantine and the ability of intracellular channel blockers to act through MCI, are currently under investigation (Nigam et al., 2023; Neureiter et al., 2024). Inhibition of VGSCs by channel blocking drugs that can access their binding sites through hydrophobic paths, including local anesthetics, antiepileptics, and antiarrhythmics, has been well characterized. Many modulatory compounds that act on KV channels, VGCCs, AMPARs, KARs, nAChRs, GABA_A_Rs, and TRP channels can access their binding site through hydrophobic paths. Channel fenestrations, a feature associated with modulation by membrane-associated drugs, have been reported in channel types in addition to those discussed here, for example, the insect gustatory receptor superfamily (Frank et al., 2024). Fenestrations appear to be a structural feature found across channel types. Channel modulation by membrane-associated drugs and neurosteroids involving fenestrations may be a nearly ubiquitous ion channel property expressed by a much wider range of channel types than we are aware.

Examination of the action of membrane-associated drugs on one channel type may aid investigation of drug action on other channel types. Similarly, comparison of ion channel structures (Figure 1) may aid researchers in identifying shared mechanisms of membrane-associated drug action. In addition, experimental protocols used to study modulation by membrane-associated drugs of one channel type may be generalized to other types of channels: for example, some of the experiments used to investigate MCI of NMDARs were based on experiments used to elucidate the mechanisms of action of VGSC blockers. These approaches include varying extracellular or intracellular pH to change the amount of uncharged drug in solution, site directed mutagenesis of hypothesized fenestration-lining residues, and examination of inhibition by permanently charged drugs that are less likely to associate with the membrane or act through a hydrophobic path. Application of similar approaches to study membrane-associated drug action on other channel types can help elucidate both shared and channel-specific inhibitory mechanisms. One important difference between the action of local anesthetics on VGSCs and MCI by channel blocking drugs of NMDARs is the state dependence of block. NMDARs must be open for channel blocking drugs to bind via either hydrophilic or hydrophobic paths. In contrast, VGSC channel blocking drugs can access hydrophobic paths to reach their binding sites when the channel is closed and have the highest affinity for inactivated channels. Further similarities and differences in the features of channel inhibition through hydrophobic paths can be revealed by performing comparable experiments on other channel types.

Currently there are no effective pharmacological therapies for most of the debilitating diseases associated with channelopathies and, of particular relevance here, fenestropathies (Gamal El-Din and Lenaeus, 2022). It is therefore essential to fully investigate the mechanisms of action of drugs that modulate channel function. Drugs that act via hydrophobic paths have specific features that distinguish them from drugs that act through other inhibitory mechanisms, and that may be clinically useful. One such feature is relatively weak voltage dependence, a consequence of voltage-independent binding of uncharged drug to the channel, in contrast to binding of charged drug within the voltage field during traditional block. It may be possible to leverage this characteristic to develop drugs that are effective even on cells that are pathologically depolarized, a clinically relevant condition.

In addition, membrane-associated modulatory drugs may be useful for targeting specific channel subtypes across a wide variety of channel families. The residues that line the path of access and the binding site of drugs that act via traditional channel block are frequently highly conserved. Membrane-associated drugs, however, may be more channel subtype-selective because they access the channel via fenestrations that may be lined by residues that are more weakly conserved and thus differ between subtypes. The unique pharmacological features of membrane-associated drugs may be useful both experimentally, providing insights into channel structure and function, and clinically, enabling development of superior pharmaceuticals for modulating ion channel function.
